# Hybrid integration of III-V semiconductor lasers on silicon waveguides using optofluidic microbubble manipulation

**DOI:** 10.1038/srep29841

**Published:** 2016-07-19

**Authors:** Youngho Jung, Jaeho Shim, Kyungmook Kwon, Jong-Bum You, Kyunghan Choi, Kyoungsik Yu

**Affiliations:** 1 School of Electrical Engineering, Korea Advanced Institute of Science and Technology (KAIST), Daejeon 34141, Korea

## Abstract

Optofluidic manipulation mechanisms have been successfully applied to micro/nano-scale assembly and handling applications in biophysics, electronics, and photonics. Here, we extend the laser-based optofluidic microbubble manipulation technique to achieve hybrid integration of compound semiconductor microdisk lasers on the silicon photonic circuit platform. The microscale compound semiconductor block trapped on the microbubble surface can be precisely assembled on a desired position using photothermocapillary convective flows induced by focused laser beam illumination. Strong light absorption within the micro-scale compound semiconductor object allows real-time and on-demand microbubble generation. After the assembly process, we verify that electromagnetic radiation from the optically-pumped InGaAsP microdisk laser can be efficiently coupled to the single-mode silicon waveguide through vertical evanescent coupling. Our simple and accurate microbubble-based manipulation technique may provide a new pathway for realizing high precision fluidic assembly schemes for heterogeneously integrated photonic/electronic platforms as well as microelectromechanical systems.

Silicon photonics is gaining significant momentum in chip-scale integration of photonics and electronics for high-performance low-power on-chip communication systems due to its compatibility with existing mass-producible fabrication processes^1^. Despite many studies^2^, however, efficient light emission directly from the silicon material has not yet been successfully achieved because of its low radiative recombination rate mainly resulting from the indirect band gap of silicon. Hybrid/heterogeneous integration of III-V compound semiconductor light sources onto the silicon photonics chips have therefore attracted considerable interest for practical implementation of chip-scale optical interconnect systems. Representative examples include hetero-epitaxial direct growth of compound semiconductor on silicon^3^,^4^ and wafer-level bonding^5^,^6^. However, because of large mismatches in lattice constants and thermal expansion coefficients between multiple semiconductor materials, large-area epitaxial growth of compound semiconductor layers on the silicon substrate might have practical limitations in terms of seamless monolithic integration in the near future. Wafer-scale bonding typically requires superb cleanliness and surface flatness down to the atomic scale, which is difficult to obtain with a high yield for large-area substrates. Due to such stringent requirements, they still present various practical issues in the process-level reliability and fabrication cost.

An obvious alternative approach for heterogeneously integrated electronic and photonic circuits is the so-called ‘pick-up and placement’ techniques with, for example, micro-grippers^7^,^8^ or stamp-assisted transfer printing^9^,^10^, which can provide simple solution for compound semiconductor materials integration on the silicon photonics platform. As the object dimension decreases to the micrometer scale for miniaturization of active semiconductor optoelectronic devices, however, it is not always easy to capture and place such small objects with required precision. For example, relatively large misalignment tolerances of the stamp-assisted transfer printing, typically on the order of one micrometer^9^, may lead to unacceptable optical coupling losses to/from the access waveguides. Inevitable physical contacts during the transfer and assembly processes might also cause detrimental damages and contamination on the contact areas.

Another strategy for the pick-and-place approach is to employ manipulation mechanisms without any physical contact to the object under fluidic environments. Well-known examples include dielectrophoresis^11^,^12^,^13^,^14^, optical tweezing^15^,^16^, and convection-based microparticle manipulation^17^,^18^,^19^. Dielectrophoretic force, which can be applied to polarizable objects with spatially non-uniform electric field profiles, has been widely used in a number of applications^11^,^12^,^13^,^14^. It offers a convenient way for accurate capture, separation, and accumulation of microscale objects, but also requires additional conducting electrode structures to generate non-uniform electric field gradients. The optical tweezer based on the electromagnetic radiation pressure has also attracted much attention as a potential manipulation tool for its accuracy and relatively simple configurations^15^,^16^. The main drawback of the optical tweezer manipulation techniques, however, is that high optical powers are typically required even for small objects, and this might cause radiation and/or thermal damages to the trapped objects. Furthermore, microscale objects placed on a flat surface with large contact areas (e.g., a disk or slab rather than a sphere) are difficult to grab and manipulate with such optical tweezing techniques because van der Waals and friction forces are usually much stronger than the optical radiation pressure^19^.

In this paper, we propose and demonstrate a laser-based optofluidic manipulation technique with on-demand and *in-situ* photothermal generation of a microbubble on the light-absorbing micro-object for pick-and-place assembly of compound semiconductor materials on the silicon photonics chip in aqueous solution. Figure 1 schematically illustrates laser-based photothermal generation of optofluidic flows, more specifically photothermocapillary convective flows around the microbubble, for trapping and manipulation of microscale objects in liquid environments. Although photothermal effects allow remote and efficient delivery of heat energy through electromagnetic radiation absorption at the manipulation objects, the conventional microbubble-based optofluidic manipulation techniques^17^,^18^,^19^ require an additional light-absorbing layer that converts the incident light energy into heat for microbubble generation. The photothermal conversion efficiency can be further enhanced by plasmon-based absorbing structures^20^, but they typically require modifications to the manipulation objects. Optoelectronic tweezers and dielectrophoretic manipulation techniques also need additional conducting electrode structures to generate electric field gradients^11^,^12^,^13^,^14^. For integrated photonics applications, however, such extra light-absorbing layers/structures might hinder efficient light coupling to/from the optical waveguides, and require additional fabrication process steps and footprints. When the microscale objects themselves have a relatively high photothermal conversion efficiency with a large electromagnetic absorption coefficient, additional photo-absorbing layers/structures would not be necessary for real-time *in-situ* microbubble generation. When a focused laser beam gets absorbed by the microscale semiconductor object and raises its local temperature beyond the boiling point of the surrounding liquid as depicted in Fig. 1a, an air bubble is generated on the object surface (Fig. 1b). After microbubble formation, the surface tension gradient around the bubble surface induced by non-uniform temperature distribution gives rise to the thermocapillary convective flow. When the laser heating spot is moved to an off-center position within the microscale light-absorbing object, the spatial symmetry in the temperature distribution and fluid flow profiles will be broken, and the photothermal convection flow near the bubble can levitate and mobilize the object (Fig. 1c). The trapped object can be dragged in any lateral directions by moving the laser heating spot and/or the substrate, and can also be precisely dropped on the substrate at the desired location by simply turning the laser illumination off. By adapting the suggested optofluidic microbubble manipulation method, we experimentally demonstrate heterogeneous assembly of III-V compound semiconductor materials onto the silicon-on-insulator (SOI) photonic integrated circuit platform. By comparing the lasing performance of the semiconductor microdisk lasers before and after the manipulation and assembly process, we verify that the semiconductor materials and the microdisk cavity structures are not damaged.

## Results

### Optofluidic thermocapillary convective flow around the microbubble

To obtain enough force for microscale object manipulation in fluids, we employ the combination of the photothermal and thermocapillary effects induced by the focused near-infrared laser beam illumination. The thermocapillary convective flow occurs when the spatial variation of surface tension around a liquid/air phase interface arises from inhomogeneous temperature distribution^17^,^18^,^19^. The shear stress resulting from the surface tension gradient drives the thermocapillary convective flow, and can be used to trap and move optically absorbent micro/nano-particles trapped on microbubbles. In this section, we study real-time microbubble generation on the compound semiconductor surface as well as the photothermocapillary force around the bubble using numerical simulations.

Since the semiconductor material (~350 nm thick InGaAsP/InGaAs in our experimental demonstration) efficiently absorbs the near-infrared manipulation light beam whose center frequency is higher than the semiconductor band gap energy, the additional light absorption layers for photothermal conversion^17^,^18^,^19^,^20^ are not necessary. According to the numerical simulations considering the measured absorption coefficient of 5200 cm^−1^ for the InGaAsP material at a wavelength of 975 nm used in our experiments, approximately 65% of the incident optical power can be absorbed within the semiconductor object before bubble generation, signifying the efficiency of light-to-heat energy conversion through absorption. With such an absorption level, for example, the top surface temperature can be raised up to 100 °C and 130 °C for a cylindrical microdisk object with a diameter of 10 μm and a thickness of 350 nm when the incident laser power is 7.35 mW and 10 mW, respectively. Consequently, a thin semiconductor block can act as an efficient, moving heat source to generate a microscale air bubble and at the same time to create the gradients of temperature and surface tension distribution.

Numerical simulations based on the finite element method (FEM) were performed to estimate the incident optical power levels for bubble generation through boiling as well as to understand the thermocapillary convective flow around the microbubble on the microdisk surface. Figure 2a plots the required input laser power to reach the boiling point of water (100 °C) for microbubble generation with respect to the compound semiconductor microdisk size. We assumed that the laser beam was focused on the microdisk surface and the diameter of the Gaussian beam waist was 1.8 μm. The semiconductor microdisk was assumed to be placed on the 500 nm-thick SiO_2_ layer on top of the silicon substrate. The minimum power level initially increases with the object diameter because of the increased heat capacity, and then eventually saturates due to thermal diffusion. Regardless of the microdisk diameter, microbubbles can be formed above the incident optical power of 8 mW, which is sufficiently low to prevent possible degradation of active semiconductor material according to our experimental results. It is also interesting to verify whether enough electromagnetic energy can be still transferred to the object after microbubble formation. The color plot inside the microbubble area in Fig. 2b describes the estimated electric field intensity (|E|^2^) distribution, showing the laser beam propagation inside the bubble region. This result was obtained from the finite-difference time-domain (FDTD) method, and indicates that the semiconductor object can still absorb approximately 60% of the incident laser power even after the bubble is generated (More details can be found in Supplementary Fig. S1). This also means that the microbubble can be sustained under the laser beam illumination. When the laser beam is turned off, however, the microdisk temperature decreases quickly due to thermal diffusion into water, and the corresponding thermocapillary force also decreases as the microbubble shrinks.

To further analyze the thermocapillary flow, the boundary condition at the water/air phase interface, *η*(*∂u*/*∂**n***) = *γ(∂T*/*∂**t**)*, is adopted to consider the effects of the temperature and surface tension gradients, where *η* is the dynamic viscosity, *u* is the fluid velocity vector, *γ* is the temperature derivative of the surface tension, *T* is the temperature, and ***n** (**t***) is the unit vector normal (tangential) to the water/air interface^21^. This boundary condition implies that the shear stress on the interface is proportional to the temperature gradient. The no-slip boundary condition is also used at the solid/water interfaces to simulate the fluid velocity distribution (shown with arrows outside the bubble region in Fig. 2b). The total simulation size (100 μm × 100 μm) is large enough not to introduce any artificial boundary effects on the temperature profile and flow dynamics. The initial temperature is set at room temperature. From the FEM simulation results, when the microdisk is sufficiently heated, the interfacial force in the tangential direction is induced around the microbubble as shown in Fig. 2b. The thermocapillary force along the vertical direction is strong enough to overcome the gravity on the semiconductor block used in the experiments (see Fig. S2 and Supplementary Information for details). Consequently, the thermocapillary flow can capture and entrain the semiconductor block attached to the microbubble. Although the exact shape and size of the microbubble generated on the semiconductor object during the actual experiments might be different from what we have assumed in our calculation, our first-order analysis elucidates the origin of the bubble formation and manipulation force using the photothermal effects from the focused laser beam.

### Optofluidic thermocapillary manipulation

Real-time optofluidic manipulation of a microbubble allows agile translation of micro/nanoscale objects in aqueous solution. The details of the sample fabrication and optofluidic manipulation setup are described in the Methods section and Supplementary Information (Figs S3 and 4). Figure 3a shows an example of trapping and manipulation/translation of a semiconductor microdisk plate on the silicon oxide layer. With sufficient laser input powers (typically ~10 mW in our experiments), an air bubble is generated at the interface between the microdisk and water (Fig. 3a, *t* = 0s). The microdisk is kept adhered to the water/air microbubble interface by the thermocapillary convective flow as mentioned in Fig. 2b. When the laser heating spot is moved away from the microdisk center, the microbubble deforms from its initial hemispherical shape and follows the laser beam toward the temperature gradient to the warmer area. As a result, the trapped microdisk can be dragged on the substrate plane, following the movement of the microbubble (and therefore the laser beam) as shown in Fig. 3a and Movie 1 in Supplementary Information. The experiment results show that the translation velocity can also be manipulated by controlling the laser scanning speed and/or the sample stage speed. Once the manipulation laser beam is turned off, the microbubble shrinks and collapses because of temperature decrease and the corresponding Laplace pressure, and finally the semiconductor microdisk lands on the substrate. As shown in Fig. 3b and Movie 2 in Supplementary Information, the orientation of a rectangular slab object can be also manipulated with the proposed manipulation method by generating the microbubble on only one side of the non-circular object. We believe that such a scheme can be further applied for assembly and integration of micro/nanoscale tubes^22^,^23^, wires^16^,^24^, and ribbons^9^,^25^ made of light-absorbing materials. More manipulation results can be found in Supplementary Fig. S5. The manipulation and registration accuracy of less than one micrometer was also obtained as explained in Supplementary Table S2.

Another important factor for the suggested microbubble-based manipulation method is the laser power level because the light absorbing object can be damaged or destroyed by intense laser illumination (e.g., >~15 mW in our experiments, see Supplementary Fig. S6). To prevent such damages during the manipulation/assembly process, the incident optical power range was maintained between 8 and 12 mW in our experiments.

## Discussion

Based on the proposed optofluidic manipulation technique, the microdisk lasers can be assembled on the SOI-based optical waveguide platform without significantly affecting the optical properties of the resonant mode. By using an additional visible light source and a charge-coupled device (CCD) camera to visualize the semiconductor microdisk and the silicon waveguide in real-time, the semiconductor microdisk can be aligned with the silicon single-mode waveguide. Figure 4a,b schematically show the optical characterization configuration after an InGaAsP microdisk laser is assembled on a silicon rib waveguide. The typical rib waveguide with a silicon layer thickness of 250 nm, an etch depth of 150 nm, and a waveguide width of 500 nm was employed, and a ~200 nm thick SiO_2_ layer was deposited on top of the whole chip for passivation. A grating coupler was placed at the end of the waveguide. Because of a large refractive index contrast between InGaAsP and SiO_2_ materials (*n*_*InGaAsP*_ = 3.5, *n*_SiO2_ = 1.4), the lasing modes (e.g., whispering gallery modes in the microdisk resonator) can be tightly confined in the optical gain material when the SiO_2_ spacer layer is sufficiently thick. The SiO_2_ layer also plays an important role in reducing the manipulation laser power for *in-situ* microbubble generation due to its low thermal conductivity (1.4 W·m^−1^K^−1^) compared to silicon (149 W·m^−1^K^−1^). For experimental verification, the assembled semiconductor microdisk structure is optically pumped at room temperature using a pulsed 1064-nm-wavelength semiconductor laser diode with a 10 ns pulse width and 200 kHz repetition rate (0.2% duty cycle). The pump laser beam was focused on the microdisk surface through a microscope objective with a numerical aperture of 0.42 and a beam spot diameter of approximately 4.2 μm. The semiconductor microdisk optically pumped from the surface normal direction can reach the lasing condition, and then its output radiation was evanescently coupled to the silicon waveguide. The optical output signals from the microdisk laser and the grating coupler were simultaneously measured by an infrared spectrometer and an optical spectrum analyzer, respectively. This vertical coupling approach has several advantages over the lateral coupling configuration, since it does not require high-resolution lithography to define a narrow sub-micron coupling gap between the waveguide and the laser resonator. In addition, the coupling strength between the microdisk resonator and the silicon waveguide is relatively insensitive to small lateral alignment deviations^26^,^27^.

To assess possible damages in the resonator structure and/or semiconductor materials during the optofluidic assembly process, we measured the output light intensity curves for ~8 μm diameter microdisk lasers before releasing from the InP substrate (square symbols), after transferring on the SiO_2_/Si layer without any microbubble-based manipulation processes (circles), and after optofluidic assembly on the silicon rib waveguide (triangles). The measurement results shown in Fig. 4c indicate that the threshold pump powers for such three cases are ~1.2 mW, ~2.2 mW, and ~2.3 mW, respectively. The absorbed threshold pump powers for the microdisk laser placed on the SiO_2_ layer is similar to that of the microdisk laser assembled on the waveguide, implying that the microdisk structure is not damaged during the manipulation and assembly processes. Due to light leakage into the SiO_2_ layer and the silicon rib waveguide, however, the resonator performances are inevitably degraded when compared to the unreleased microdisk lasers on the InP pedestal. The full width at half maximum (Δ*λ*) of emission spectrum peak near the lasing threshold is measured to be ~0.45 nm, which corresponds to the total loaded quality factor (*λ/Δλ*) of ~3500. Figure 4d shows an optical microscope image of a microdisk laser assembled on the silicon rib waveguide and its lasing spectrum measured from the grating coupler.

## Conclusions

In conclusion, optofluidic manipulation and assembly of microscale compound semiconductor materials for silicon photonic integrated circuits has been experimentally demonstrated using laser-induced photothermal microbubble generation and photothermocapillary flows. The semiconductor microdisk lasers with diameters ranging from 5 to 20 μm are assembled on top of the silicon rib waveguides with less than one micrometer precision. By taking advantage of the vertical evanescent coupling process, electromagnetic radiation from the optically pumped microdisk laser can be efficiently transferred to the single-mode silicon waveguide and routed to other integrated optical components. Our accurate and simple optofluidic pick-and-place method facilitates the development of high precision micro/nano-assembly schemes for heterogeneously integrated photonic/electronic platforms as well as microelectromechanical systems^7^,^28^. An important advantage of the proposed technique is that it does not require additional structures^17^,^18^,^19^ and electric sources^11^,^12^,^13^,^14^ for optofluidic manipulation as long as the micro-objects strongly absorb the manipulation beam. In addition to the silicon photonics optoelectronic integration, basic translation and rotation techniques for small semiconducting objects and light-absorbing blocks can also be applied to build a wide range of electronic and photonic devices/systems, such as efficient detectors^22^, single electron transistor^23^, and light-emitting diodes^24^.

## Methods

### Preparation of semiconductor microdisk lasers

For semiconductor microdisk laser fabrication, multiple compound semiconductor epitaxial layers were grown by metal-organic chemical vapor deposition on an InP substrate. The InGaAsP layer has a photoluminescence peak at around 1550 nm at room temperature. The details of the layer structure are shown in Supplementary Table S1. Typical photo-lithography techniques were used to define circular microdisk patterns with diameters ranging from 5 to 20 μm. The released semiconductor microdisks were immersed in deionized water and transferred to the silicon substrate using a syringe. Fig. S3 of Supplementary Information shows the scanning electron micrographs (SEMs) of the semiconductor microdisks whose smooth sidewalls help reducing the scattering losses of whispering-gallery resonant modes. The detailed fabrication and transfer processes can be found in Supplementary Information.

### Optofluidic thermocapillary manipulation setup

**Figure 1:**
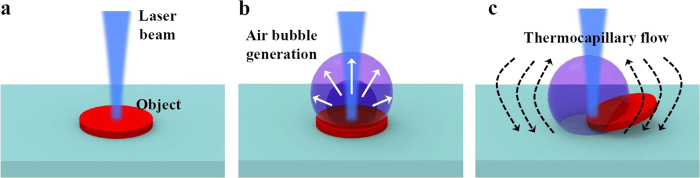
Photothermal *in-situ* microbubble formation and optofluidic flow generation for trapping and manipulation of the microscale light-absorbing object. (**a**) An optically absorbent micro-object in liquid is heated by a focused laser beam. (**b**) An air bubble is formed on the object surface when the liquid temperature near the heating spot reaches the boiling point. (**c**) The temperature and surface tension gradients produce the thermocapillary convective flow around the air bubble, on which the light-absorbing object can be trapped.

**Figure 2:**
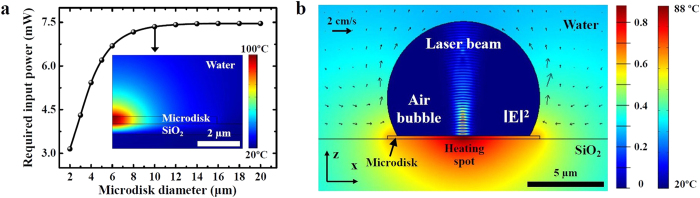
The computer simulation results for the laser-induced photothermal process. (**a**) Required laser input power to reach the boiling point of water as a function of the microdisk diameter. The inset shows a cross-sectional temperature distribution profile for the semiconductor microdisk with a diameter of 10 μm when the incident 975 nm wavelength laser power is 7.35 mW. (**b**) Thermocapillary convective flow generated around the air bubble for a 10 μm-diameter semiconductor microdisk. The arrows and surface colors outside the air bubble represent the fluid velocity and the temperature distribution profile, respectively. The surface colors inside the bubble show the ratio of the electric field intensity to the incident laser beam after bubble formation. The vector scale bar for the fluid velocity represents 2 cm/s.

**Figure 3:**
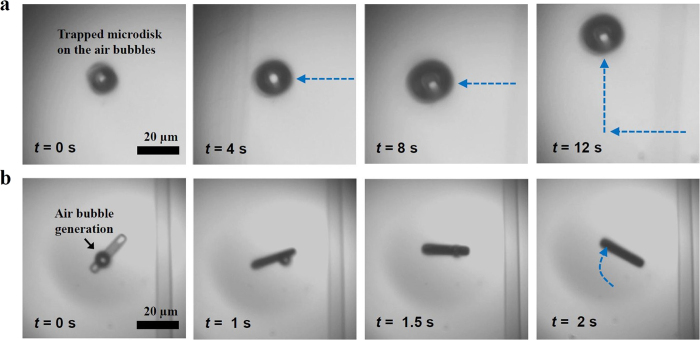
Time-sequenced optical microscope snapshots demonstrating (**a**) the translation of a cylindrical microdisk object and (**b**) the orientation control of a rectangular slab object (Movie 1 and 2 in Supplementary Information). The diameter and thickness of the microdisk is ~13 μm and 350 nm, respectively. The width and length of the rectangular object are ~4 μm and ~23 μm, respectively.

**Figure 4:**
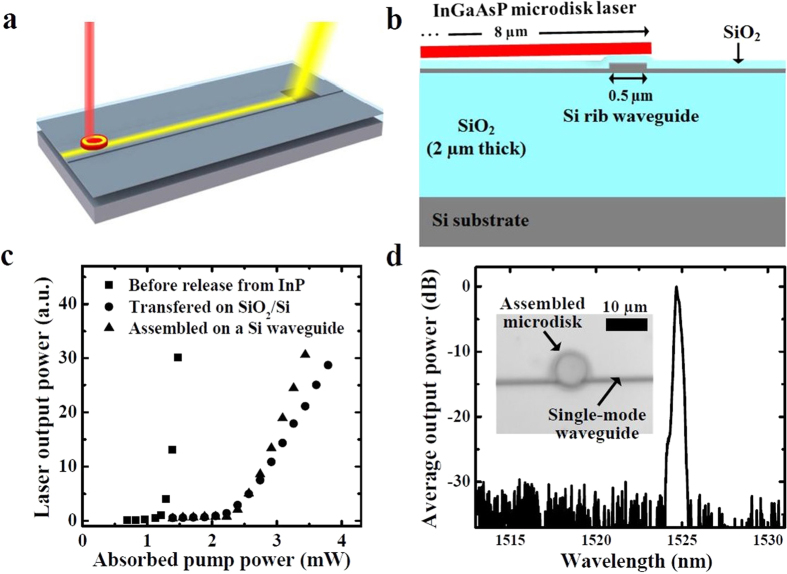
Heterogeneous integration of an InGaAsP microdisk laser on a silicon waveguide and its optical characterization results. (**a**) Perspective view and (**b**) cross-sectional view of the assembled microdisk laser. (**c**) The lasing characteristics of the microdisk lasers on the InP pedestal (before releasing), transferring on the SiO_2_/Si layer without a waveguide, and after assembly on the silicon waveguide. (**d**) A lasing spectrum example obtained from an assembled microdisk measured with the grating coupler at the end of the waveguide. The inset shows an optical microscope image of the assembled microdisk on a single-mode silicon rib waveguide.

For optofluidic thermocapillary manipulation of microscale compound semiconductor blocks in aqueous solution, a continuous-wave 975-nm-wavelength optical beam from a semiconductor laser diode was focused on the object semiconductor surface through an objective lens to locally raise its temperature and eventually generate a microbubble, thereby mobilizing the semiconductor object as schematically described in Fig. 1. The manipulation laser beam had a diameter of ~1.8 μm, and its optical power measured at the object plane in liquid ranged between 8 and 12 mW. By translating the laser beam position or the sample substrate (SOI-based silicon photonics chip with integrated waveguides and grating couplers in our experiments), the light-absorbing object (compound semiconductor microdisk) trapped by the microbubble could be laterally translated on the substrate plane. The overall experimental setup for optofluidic thermocapillary manipulation and imaging is similar to a typical upright optical microscope with an additional laser excitation port. More details about the setup can be found in Supplementary Information (Fig. S4). A simple polydimethylsiloxane well structure with a height of approximately 2 mm was used to contain the aqueous solution of semiconductor blocks.

## Additional Information

**How to cite this article**: Jung, Y. *et al*. Hybrid integration of III-V semiconductor lasers on silicon waveguides using optofluidic microbubble manipulation. *Sci. Rep.* **6**, 29841; doi: 10.1038/srep29841 (2016).

## Supplementary Material

Supplementary Information

Supplementary Movie 1

Supplementary Movie 2
