# Supplementary material for: Hybrid integration of III-V semiconductor lasers on silicon waveguides using optofluidic microbubble manipulation
Source: Sci Rep. 2016 Jul 19;6:29841. doi: 10.1038/srep29841 (PMC4949432; doi:10.1038/srep29841)
Supplement: Supplementary Information [file srep29841-s1.pdf]

Supplementary Information for

hybrid integration of III-V semiconductor lasers on silicon waveguides

using optofluidic microbubble manipulation

Youngho Jung, Jaeho Shim, Kyungmook Kwon, Jong-Bum You, Kyunghan Choi, and Kyoungsik Yu

School of Electrical Engineering, Korea Advanced Institute of Science and Technology (KAIST), Daejeon 34141, Korea

**Supplementary Figure S1**	Laser beam propagation and absorption after microbubble generation
**Supplementary Figure S2**	Thermocapillary force around the air bubble
**Supplementary Figure S3**	SEMs of a microdisk on the InP pedestal layer and a microdisk after transferring onto a SiO2/Si wafer
**Supplementary Figure S4**	Experimental setup for optofluidic thermocapillary flow manipulation using photothermal microbubble generation
**Supplementary Figure S5**	CCD images of the aligned rod-like rectangular objects
**Supplementary Figure S6**	SEM of microdisk manipulation results in the holes to check thermal and radiation damages
**Supplementary Table S1**	Epitaxial layer structure for the semiconductor microdisk fabrication
**Supplementary Table S2**	Assembly accuracy of the microdisks placed near the silicon waveguide
**Supplementary Video 1**	The translation of a cylindrical microdisk object using microbubble manipulations
**Supplementary Video 2**	The orientation control of a rectangular slab object using microbubble manipulation

Laser beam propagation and absorption after microbubble generation

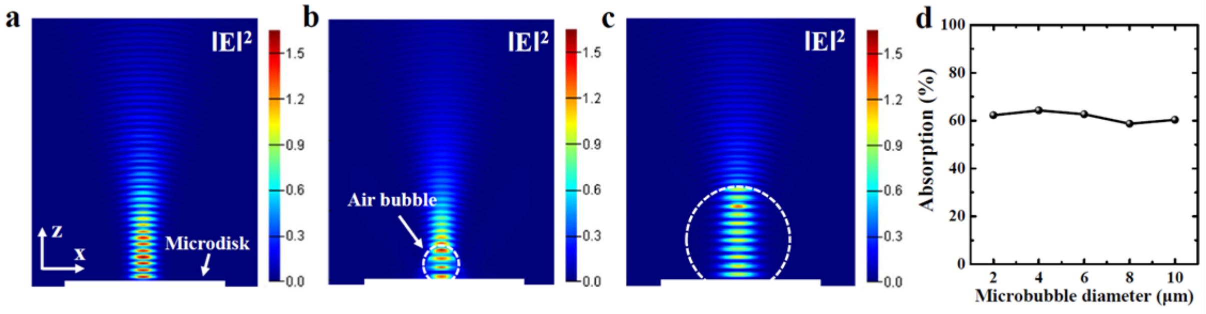

Figure S1. The electric field intensity ($|E|^2$) distributions when the microbubble diameters are (a) 0 μm (before the microbubble generation), (b) 2 μm and (c) 6 μm, showing the focused laser beam propagation after the formation of a microbubble on the semiconductor microdisk surface. The laser beam propagation for a 10 μm diameter microbubble is shown in Fig. 2b (inside of the bubble region). (d) Absorption within the InGaAsP microdisk block as a function of the microbubble diameter. We assumed that the laser beam was focused on the surface of a 10 μm diameter microdisk, and its spot size was 1.8 μm. The laser beam does not diverge significantly inside the microbubble, and the absorption ratio of over 60 % is retained regardless of the microbubble size.

Thermocapillary force

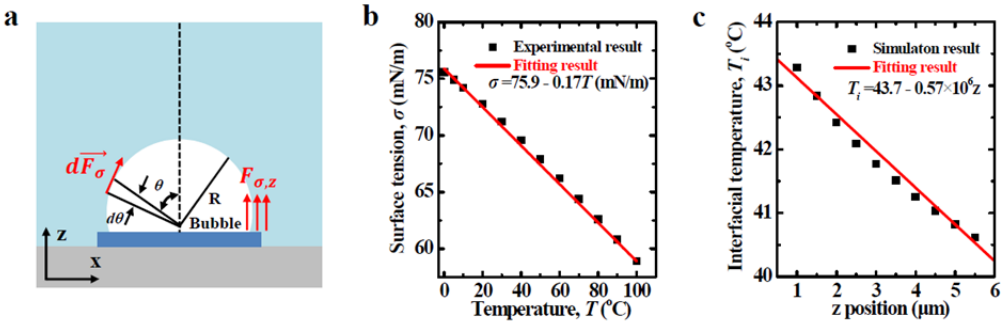

Figure S2. (a) Schematic view of the thermocapillary force around the air bubble. (b) The experimental and linear fitting results of surface tension as a function of the temperature. (c) The simulation and linear fitting results of interfacial temperature as a function of the vertical position.

Assuming negligible internal flows inside the microbubble at the steady state, the interfacial force by the shear stress at the latitude angle θ can be written as[1]

$$d\overrightarrow{F_\sigma}(\theta) = \frac{\partial \sigma(\theta)}{\partial \theta}(2\pi R \sin\theta)\, d\theta$$

where R and $\sigma(\theta)$ stand for the radius of the microbubble and surface tension, respectively, as indicated in Fig. S2a. Its vertical component ($F_{\sigma,z}$) is given by

$$F_{\sigma,z} = \int_0^{\theta_{\max}} d\overrightarrow{F_\sigma} \sin\theta \, d\theta = \frac{4}{3}\pi R^2 \left(\frac{\partial\sigma}{\partial T}\right)\left(\frac{\partial T_i}{\partial z}\right)\left(1-\cos\theta_{\max}\right)$$

where T_i indicates the interfacial temperature between the microbubble and the water. To estimate the amount of force under the experiment condition, the radius of the microbubble, R, was set to be 5 μm, and its shape was considered as being almost hemispheric (θ_{max}=101°). The experimental results of surface tension at water-air interface[2] as a function of temperature was used to approximate the surface tension gradient term ($\partial\sigma/\partial T$=0.17 mN/m) as a constant value (Fig. S2b). The interfacial temperature distribution in Fig. S2c was obtained from numerical simulations assuming that the input laser power was 8 mW and its beam spot diameter was 1.8 μm. We also assumed that the laser beam was applied at 3 μm away from the microdisk center to emulate actual experiment conditions. The air-water interfacial temperature was assumed to vary linearly with the vertical direction (z) whose fitting result is given by $T_i = 43.7-0.57\times10^6 z$. Applying the linear fitting results, the vertical thermocapillary force of ~12 nN is obtained.

Preparation of semiconductor microdisk lasers

For semiconductor microdisk fabrication, compound semiconductor epitaxial layers were grown by metal-organic chemical vapor deposition on an InP substrate. The InGaAsP bulk layer has a photoluminescence peak at around 1550 nm at room temperature. The details of the layer structure are shown in Table S1. Photo-lithography was used to define circular microdisk patterns with diameters ranging from 5 to 20 μm. The InP/InGaAsP layer was then chemically etched using bromic acid (HBr), phosphoric acid (H_3PO_4), and potassium dichromate ($K_2Cr_2O_7$) solutions[3], which preserve a photoresist layer during the etching process for reliable dimension control. After removing the photoresist mask, the InP substrate was further selectively etched with diluted hydrochloric acid (HCl) to create pedestals supporting the microdisk structures (Fig. S3a). Optical characterization results of a typical microdisk laser with a diameter of ~8 μm placed on the pedestal can be found in Fig. 4c. When further etched with HCl, the semiconductor microdisks can be released from the substrate. The released microdisks were then immersed in deionized water and transferred to the silicon substrate (PDMS well shown in Fig. S4) using a syringe. Figures S3a and S3b show the scanning electron micrographs (SEMs) of the semiconductor microdisk on the InP pedestal before the releasing process, and on

the SiO_2/Si wafer after the transfer process, respectively. For Fig. S3b, an additional 5 nm thick Pt layer was coated to reduce the charging effects from the dielectric SiO_2 layer.

Layer	Thickness
InP	5 nm
InGaAsP – Optical gain region	310 nm
InP	5 nm
InGaAs	30 nm
InP - Substrate	350 µm

Table S1. Epitaxial layer structure for the semiconductor microdisk fabrication.

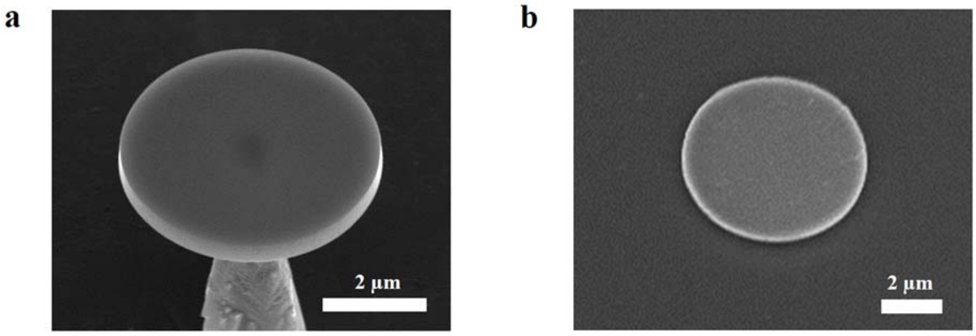

Figure S3. SEMs of (a) a microdisk on the InP pedestal layer and (b) a microdisk after transferring onto a SiO_2/Si wafer. The diameter and thickness of the microdisk are ~5 µm and ~350 nm, respectively.

Optofluidic manipulation setup

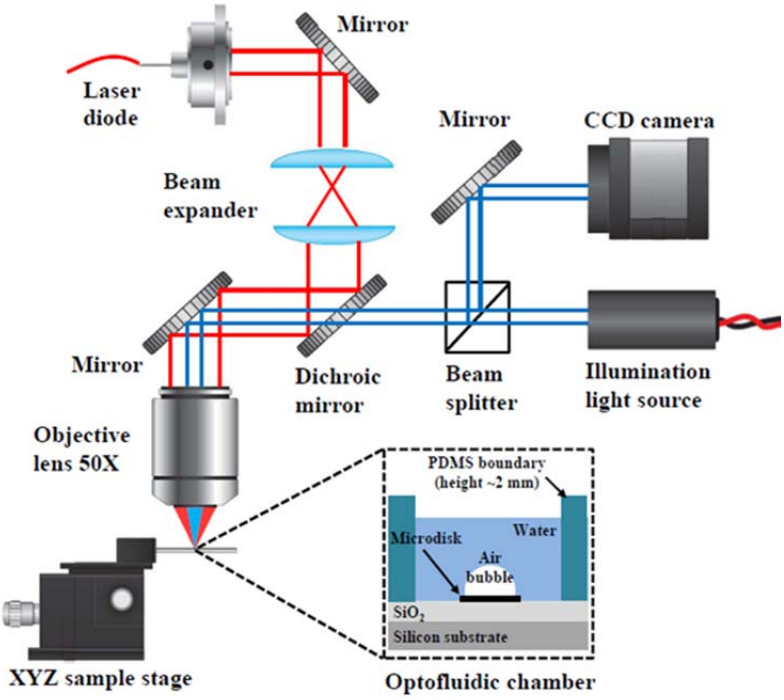

Figure S4. Experimental setup for optofluidic thermocapillary manipulation using photothermal microbubble generation.

The overall experimental setup schematically described in Fig. S4 is similar to a typical upright optical microscope with an additional laser excitation port. A visible light source was used to illuminate and visualize the microdisks and the substrate, and their optical image was projected to a charge-coupled device (CCD) camera for real-time observation. The microfluidic chamber for aqueous solution containment consists of a simple polydimethylsiloxane (PDMS) well with a ~2 mm height, as shown in the inset of Fig. S4. The semiconductor microdisks released from the InP substrate were dispersed in deionized water and transferred into this well for further optofluidic manipulation and assembly on the silicon waveguides.

Manipulation and orientation control of non-circular objects

Figure S5 illustrates examples of translation and orientation control for the rod-like objects using the suggested microbubble manipulation technique. The 350 nm thick semiconductor objects were obtained by the same fabrication procedure explained above. The width and length of the semiconductor block are ~4 µm and ~23 µm, respectively. After a microbubble is generated on one side of the rod-like object, it can be rotated by moving the laser beam in a tangential direction to the microbubble.

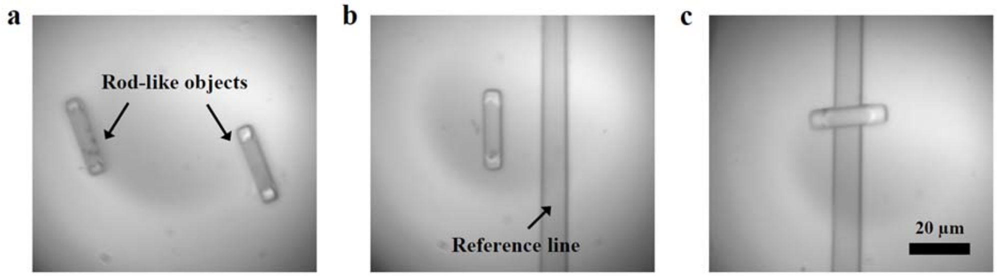

Figure S5. CCD images of the aligned rod-like rectangular objects. (a) Two rectangular objects are dragged and moved to be almost parallel to each other. The rectangular object is moved to be (b) parallel and (c) vertical to the silicon rib waveguide.

Thermal and radiation damages

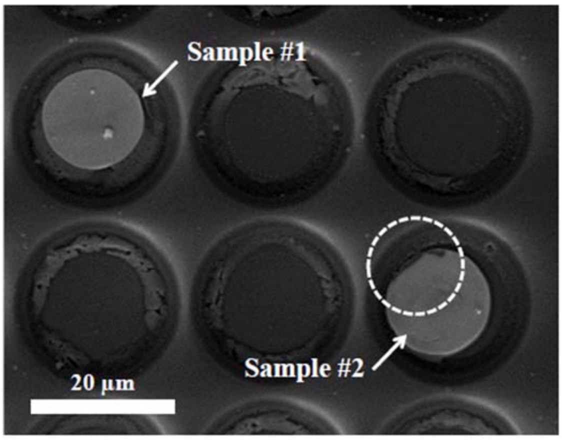

Figure S6. SEM of microdisk manipulation results in the holes. The diameters of the hole and microdisk are ~20 µm and ~14 µm, respectively.

Figure S6 shows the results of microdisk manipulation in the holes and the effect of the manipulation laser beam power. At relatively low power levels (~10 mW), there is no appearent damage to the manipulating object (Sample #1). Increasing the input beam power (up to 18 mW) results the microdisk object being destroyed during the assembly process as seen in the white circle areas of sample #2.

Manipulation accuracy

<table>
<tr><td rowspan="7">CCD image
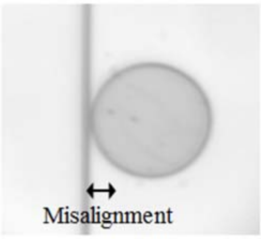
Sample #5</td><td>Sample</td><td>Diameter (μm)</td><td>Misalignment (nm)</td></tr>
<tr><td># 1</td><td>~ 5.2</td><td>~ 213</td></tr>
<tr><td># 2</td><td>~ 8.0</td><td>~ 213</td></tr>
<tr><td># 3</td><td>~ 8.9</td><td>~ 284</td></tr>
<tr><td># 4</td><td>~ 14.9</td><td>~ 355</td></tr>
<tr><td># 5</td><td>~ 16.3</td><td>~ 284</td></tr>
<tr><td>Average</td><td></td><td>269 ± 53</td></tr>
</table>

Table S2. Assembly accuracy of the microdisk placed near the silicon waveguides.

In our experiments, the manipulation accuracy can be judged by the lateral gap between the edges of the microdisk and the silicon waveguide. The lateral misalignments are measured for five different example cases with various microdisk diameters ranging from 5 to 16 μm. Average misalignment was measured to be 269 nm ± 53 nm as summarized in Table S2.

REFERENCES

1 Ghiaasiaan, S. M. *Two-phase flow, boiling, and condensation: in conventional and miniature systems*. (Cambridge University Press, 2008).

2 Vargaftik, N. B., Volkov, B. N. & Voljak, L. D. International tables of the surface tension of water. *J. Phys. Chem. Ref. Data* **12**, 817-820 (1983).

3 Park, E.-H., Kim, M.-J., Cha, J.-H. & Kwon, Y.-S. Novel high-radiance surface-emitting light emitting diode structure with circular 45 corner reflector and microlens. *Jpn. J. Appl. Phys.* **40**, 2741-2746 (2001).
